# How to examine the front of the eye

**Published:** 2019-12-17

**Authors:** Nasiru Muhammad

**Affiliations:** 1 Department of Ophthalmology, Usmanu Danfodiyo University Teaching Hospital, Sokoto, Nigeria.

**A number of common eye diseases can be diagnosed by examining the front of the eye using a torch.**

**Figure 1:**
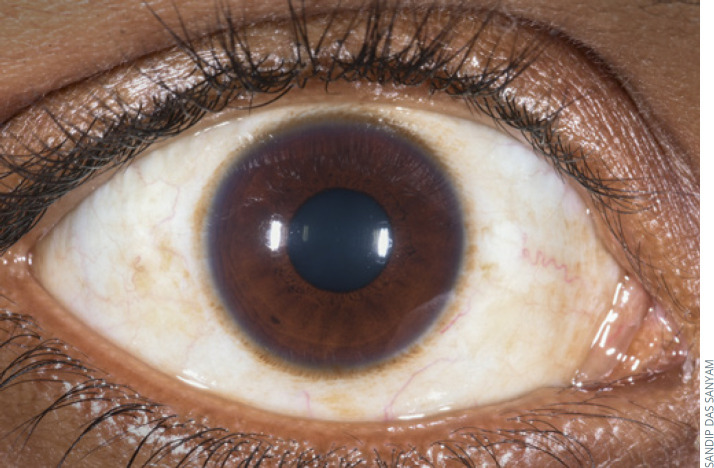
A healthy eye. The pupil is black, the white of the eye is white (not red), the eyelashes point outwards and the cornea and conjunctiva are clear.

## Basic eye examination using a torch

It is important that all health care workers know how to examine the eyes. Use of a slit lamp microscope is a gold-standard method of examining the eye but a basic examination of the front of the eye can be carried out with a torch; if a magnifying loupe is attached to the torch this is helpful but not essential. A +20 DS lens, if available, can also be used to magnify the anterior eye used in conjunction with the torch.

Figure 1 shows a healthy eye. There are four key parts to examine:

- The eyelids
- The conjunctiva
- The cornea
- The pupil

Only some common conditions, that can be seen using the above basic examination, are discussed below. All signs should be linked to the history of symptoms as this will aid the differential diagnosis. Management of specific conditions is beyond the scope of this article.

## The eyelids – do they look normal?

When examining the eyelids, check that they move normally, are in the correct position, and that there are no swellings or lumps.

**Figure 2:**
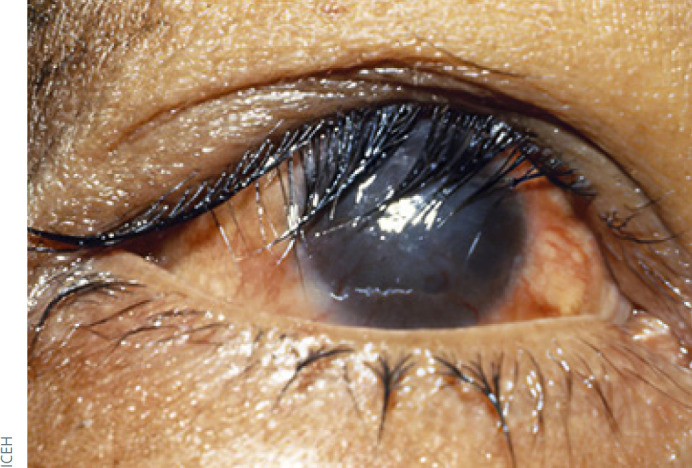
Trichiasis

**Figure 3:**
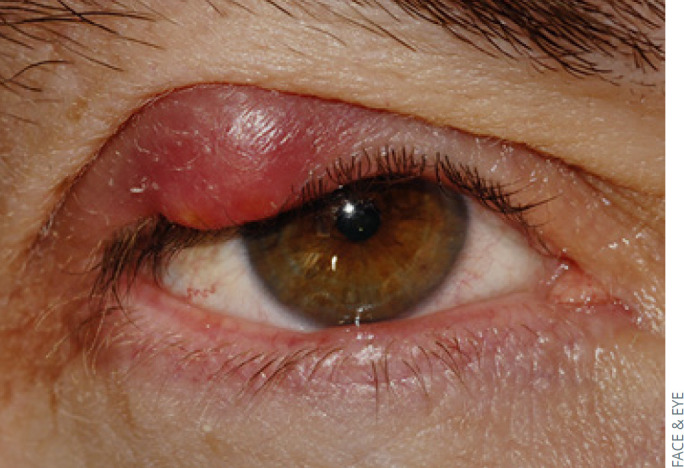
Chalazion

Check that:

- The eyelids open and close normally. Ptosis is a term used to describe drooping of the eyelids, and if they cannot close completely it is called lagophthalmos. If the eyelids cannot close, the patient is at risk of damage to the cornea.
- Neither eye is further forward than the other. When one eye protrudes further forward this is known as proptosis, which is usually a serious condition.
- The eyelashes point away from the eyeball; if they turn in on the eye this is abnormal and is called trichiasis (Figure 2). This can cause corneal scarring and blindness.
- There are no swellings or lumps on the eyelids. A swelling on the eyelid margin can be due to a cyst, called a chalazion (Figure 3), or an infection of an eyelash is called a stye.
- There is no redness or discharge at the eyelid margin, termed blepharitis.

## The conjunctiva – does the white of the eye look white?

The conjunctiva is a transparent layer which extends from the outer edge of the cornea, across the sclera (the white part of the eye) and along the insides of both eyelids. The conjunctiva contains blood vessels and, when there is a problem, the eye will often appear red.

**Figure 4:**
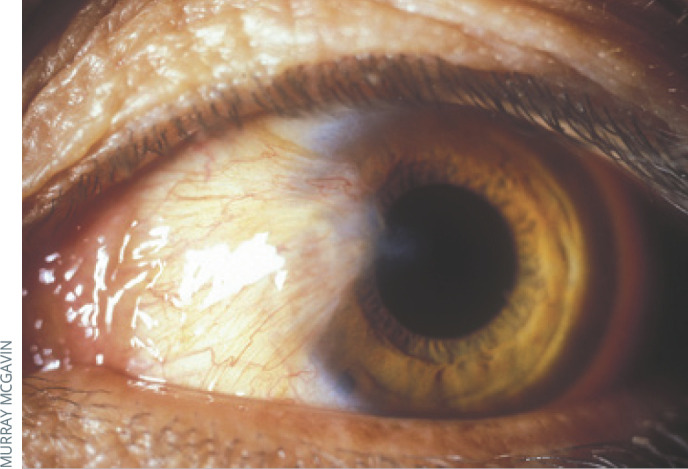
Pterygium

**Figure 5:**
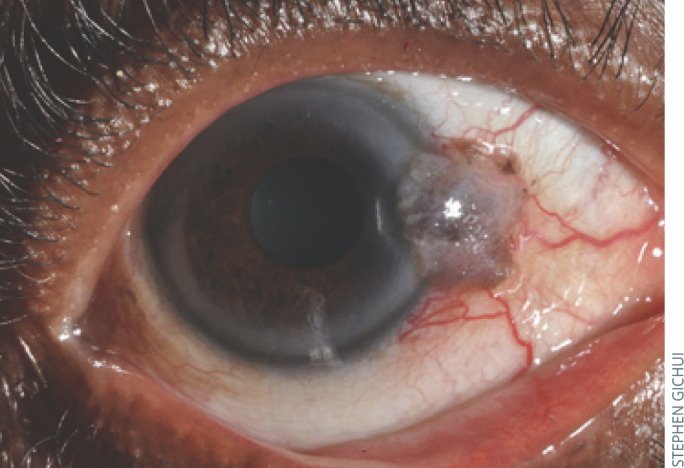
A cancerous growth on the conjunctiva

Look for:

- Redness of the conjunctiva (conjunctivitis); this might be due to infection; or allergy.
- Growths or raised fleshy areas, for example a pterygium (Figure 4) which is usually bilateral, involving the cornea; or a tumour (conjunctival carcinoma – see Figure 5);
- Foreign bodies causing irritation and often redness; it may be necessary to invert the eyelid as occasionally a foreign body is under the eyelid.

## The cornea – is it clear?

The cornea is the clear part of the front of the eye. It acts like a window and allows light to get into the eye. To examine the cornea, shine the light from the side. If there is a problem, the cornea may appear cloudy or hazy.

**Figure 6:**
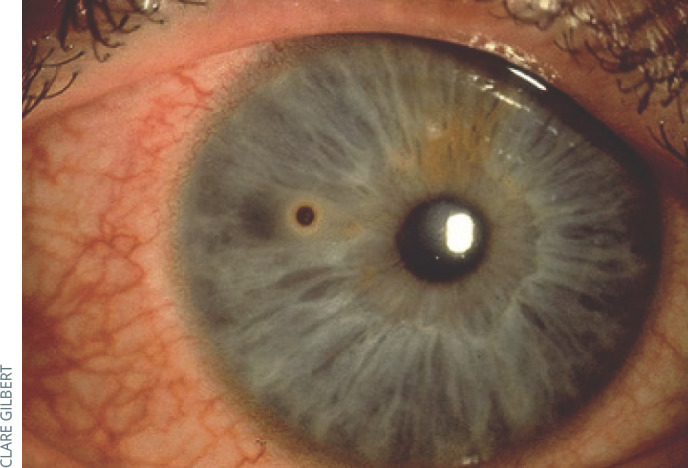
A foreign body on the cornea

**Figure 7:**
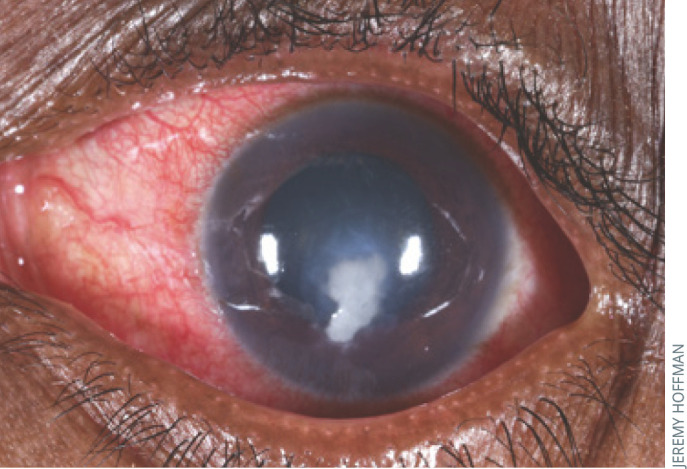
Corneal ulcer

Check that:

- There is no foreign body on the cornea; this is usually unilateral accompanied by pain or discomfort (Figure 6).
- There are no white or grey areas on the cornea; a white or grey area in a red or painful eye is likely to be a corneal ulcer (Figure 7); a white or grey area in a white, non-painful eye is likely to be a corneal scar.

## The pupil – is the pupil black and does it react to bright light?

The pupil is a round opening found in the centre of the iris, which is the coloured ring-like structure inside the eye, behind the cornea. The iris gives the eye its colour.

The pupil regulates the amount of light that goes into the eye; it is small in bright light and large in a dark room.

**The swinging torch test**

If the torch is moved from one pupil to another and back again (the swinging torch test), each pupil should become small when the light is shone at it. If this does not happen (i.e., a pupil dilates when the light is swung towards it), this may indicate a relative afferent pupillary defect (RAPD) in that eye. When RAPD is present, this suggests disease of the retina or optic nerve.

Check:

- The colour of the pupil: it should be black. A white or grey pupil may be due to cataract (opacity of the lens).
- The shape of the pupil: it should be circular. An irregular-shaped pupil may be due to injury or inflammation inside the eye (called iritis).
- That the pupil becomes small when a bright light is shone into the eye in a dark room; this indicates that the optic nerve at the back of the eye is working; if the pupil does not become small with a bright light it may be due to damage of the nerve.
- The pupils' reaction to the swinging torch test (see panel above).
- The red reflex – see p. 54.

A careful examination of the front of the eye, using a torch, can help the eye health worker identify abnormalities which can lead to a diagnosis and assist in deciding the best management for the patient.

